# Imperforate Anus and Rectourethral Fistula in a Female

**DOI:** 10.1055/s-0039-1692411

**Published:** 2019-06-27

**Authors:** Hira Ahmad, Devin R. Halleran, Daniel Dajusta, Katherine McCracken, Marc A. Levitt, Richard J. Wood

**Affiliations:** 1 Center for Colorectal and Pelvic Reconstruction, Nationwide Children’s Hospital, Columbus, Ohio

**Keywords:** colorectal surgery, rectourethral fistula, anorectal malformation, colorectal and pelvic reconstruction

## Abstract

Anorectal malformations (ARM) are complex, heterogeneous disorders and in females the most common anomaly is imperforate anus with a rectovestibular fistula. We describe a malformation not previously encountered in the literature: imperforate anus associated with a normal urethra, normal vagina, but with a recto urethral fistula. Rectourethral fistula in a female is an extremely rare ARM. Precise workup is required to clarify the anatomy for operative planning.

## Background

Anorectal malformations (ARM) are complex, heterogeneous disorders and in females the most common anomaly is imperforate anus with a rectovestibular fistula. We describe a malformation not previously encountered in the literature: imperforate anus associated with a normal urethra, normal vagina, but with a rectourethral fistula. Herein we discuss the patient's preoperative evaluation and management.

## Presentation

**Fig. 1:**
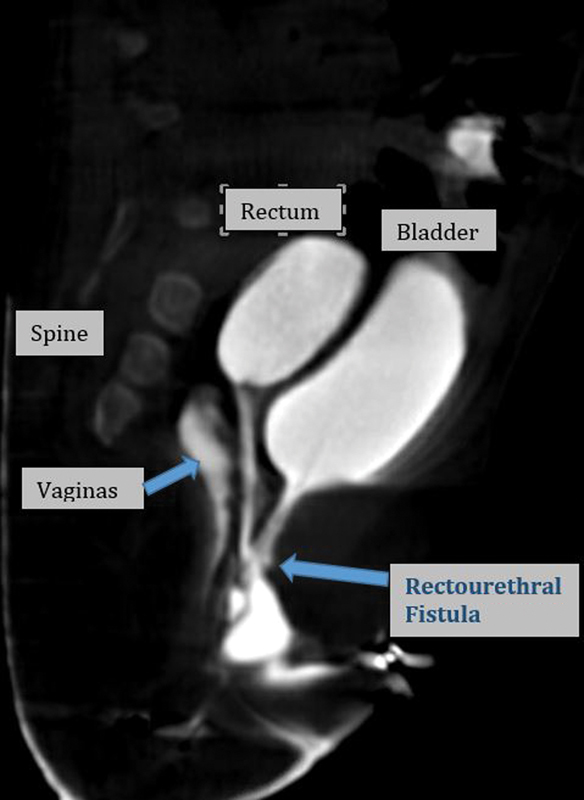
Rectal fistula inserting in the posterior urethra.

**Fig. 2:**
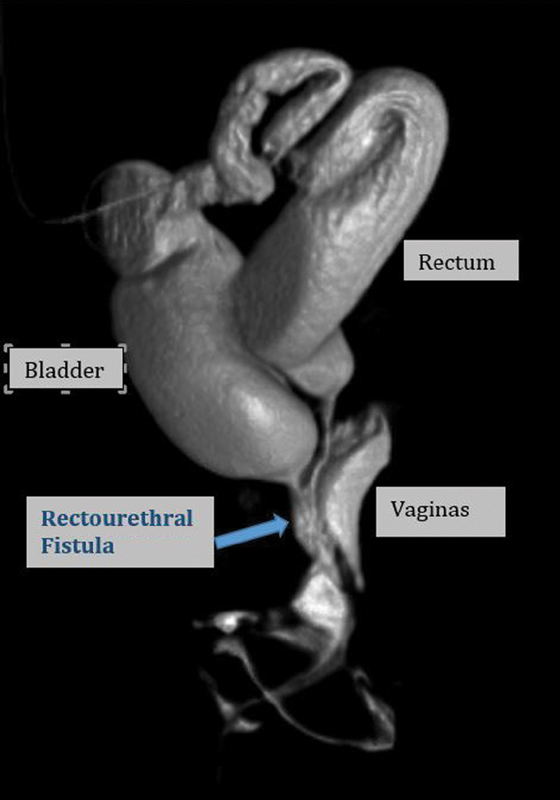
Rectourethral fistula, normal vagina as shown on 3D contrast study.

A full term newborn girl with imperforate anus underwent a laparoscopic assisted colostomy and mucous fistula at an outside facility. Testing for VACTERL association was significant for an atrial septal defect, solitary left kidney with vesicoureteral reflux, occult spinal dysraphism with tethered cord, caudal syrinx, a lateral sacral ratio of 0.57, and a didelphic uterus. She presented to our clinic at one month of age for evaluation. The findings on a contrast study showed a normal urethral meatus with urethral length of 4.4 cm (from meatus to bladder neck), colonic fistula length of 2.8 cm with insertion into the posterior urethra, and two vaginas joining into a single introitus ( Figs. 1 and 2 ). Her radionuclear renal scan confirmed a solitary left kidney with duplex configuration, with no significant hydronephrosis. She underwent an examination under anesthesia with the colorectal, urology, and gynecology teams. The colorectal examination revealed no anal opening in the perineum. She had good contraction of the sphincter complex and a well-developed anal dimple. Vaginoscopy demonstrated a longitudinal vaginal septum. Cystoscopic examination was significant for a fistula of 8 mm size from the urethral meatus on the posterior wall of the urethra. At this location, there was a split anteriorly going into the bladder (the normal urethra) and posteriorly into the rectourethral fistula going into the rectum.

We took the patient to the operating room for definitive repair of her imperforate anus with rectourethral fistula. A urinary catheter was placed cystoscopically through the fistula into the colon and another one into the bladder. We then mobilized the distal colon and rectum via posterior sagittal incision and laparotomy, and dissected the rectourethral fistula behind the bladder close to its insertion into the urethra. Laparoscopy had been attempted, however, significant intra-abdominal adhesions were encountered and we felt the laparoscopic approach could not be safely achieved. With the fistula isolated, we placed a suture ligature into the fistulous tract. We transected the rectum just above the tract to free the rectum for the rectal pull through. We pulled the catheter that had been placed into the fistula along with the fistulous tract and everted it through the urethra under cystoscope guidance. Once everted, the fistulous tract was excised and the defect in the urethra closed using 5–0 polydioxanone (PDS) suture. We then completed the pull-through. A suprapubic tube was placed. The patient tolerated the procedure well and was discharged home on postoperative day 5.

The patient underwent an examination under anesthesia and cystoscopy one month after the surgery and the urethra and anoplasty was found to be well healed. She emptied her bladder well and the suprapubic tube was removed. The patient did not require dilations post operatively, with the Hegar size 14 on examination, and no structure has developed now six months later.

## Discussion

Anorectal malformations (ARM) are congenital anomalies that occur in approximately 1 in 5,000 live births. 1 2 These malformations include a wide spectrum of defects affecting both males and females and may involve the gastrointestinal (anus and rectum) and genitourinary tracts. Anorectal malformations can range from mild anal anomalies to complex cloacal defects in females. 2 Approximately 50% of patients have an associated genitourinary defect. 1 3 4 5 Accurate diagnosis is of paramount importance to guide surgical strategy and prevent urological complications.

The most common anorectal malformation in a female patient is rectovestibular fistula. 6 On perineal examination, such a patient has a normal urethra, normal vagina, and another orifice (rectal fistula in the vestibule). 7 8 Herein we present a case of an extremely rare anorectal malformation of rectourethral fistula in a female that, to our knowledge, has not been described previously in the literature. In review of senior authors' series of over 1,000 primary ARM patients, this is the only such case. The key decisions in the management of this patient focused on maximizing urinary and rectal continence potential and promoting long-term renal health. To aid in that decision making, accurate and precise measurement of critical pelvic structures prior to reconstruction was essential.

In our patient, as seen in Figs. 1 and 2 , preoperative imaging showed a normal urethral length, and a rectourethral fistula. This allowed for better operative planning in combination with the urology team. With a tethered cord and lateral sacral ratio of 0.57, we predict the future continence of this patient to be only moderate. Our patient underwent successful takedown of rectourethral fistula and pull through of the rectum. Identifying the fistula using cystoscopy and inverting it into the urethra with urethral repair were key steps to solving this anatomic challenge.

## Conclusion

Anorectal malformations are a heterogenous group of disorders with varying presentations and prognosis. Rectourethral fistula in a female is an extremely rare ARM. Precise workup is required to clarify the anatomy for operative planning.

## Notes

New Insights and the Importance for the Pediatric Surgeon Rectourethral fistula in a female is an extremely rare anorectal malformation (ARM). Accurate diagnosis is of paramount importance in ARM patients to guide surgical strategy and prevent urological complications.
